# Benzyl­triethyl­ammonium tetra­chlorido­ferrate(III)

**DOI:** 10.1107/S1600536812023008

**Published:** 2012-05-26

**Authors:** Lei Jin

**Affiliations:** a College of Chemistry and Chemical Engineering, Southeast University, Nanjing 210096, People’s Republic of China

## Abstract

In the title mol­ecular salt, (C_13_H_22_N)[FeCl_4_], three of the chloride ions of the tetra­hedral Fe^III^-containing anion are disordered over two orientations in a 0.656 (11):0.344 (11) ratio. In the crystal, there are no identifiable directional inter­actions between cations and anions except for Coulombic forces.

## Related literature

For background to mol­ecular–ionic ferroelectrics, see: Zhang *et al.* (2010 ▶).
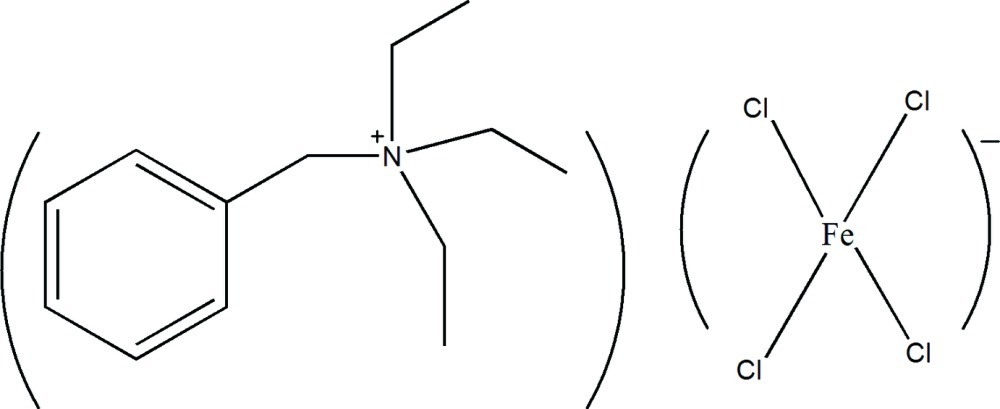


## Experimental

### 

#### Crystal data

(C_13_H_22_N)[FeCl_4_]
*M* *_r_* = 389.97
Orthorhombic, 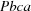

*a* = 15.514 (3) Å
*b* = 15.021 (3) Å
*c* = 16.155 (3) Å
*V* = 3764.7 (13) Å^3^
*Z* = 8
Mo *K*α radiation
μ = 1.36 mm^−1^
*T* = 293 K
0.26 × 0.24 × 0.20 mm

#### Data collection

Rigaku Mercury2 CCD diffractometer
Absorption correction: multi-scan (*CrystalClear*; Rigaku, 2005 ▶) *T*_min_ = 0.655, *T*_max_ = 0.734
32945 measured reflections
3691 independent reflections
2364 reflections with *I* > 2σ(*I*)
*R*_int_ = 0.082

#### Refinement

*R*[*F* ^2^ > 2σ(*F* ^2^)] = 0.068
*wR*(*F* ^2^) = 0.170
*S* = 1.04
3691 reflections
204 parameters
156 restraints
H-atom parameters constrained
Δρ_max_ = 0.73 e Å^−3^
Δρ_min_ = −0.38 e Å^−3^

### 

Data collection: *CrystalClear* (Rigaku, 2005 ▶); cell refinement: *CrystalClear*; data reduction: *CrystalClear*; program(s) used to solve structure: *SHELXS97* (Sheldrick, 2008 ▶); program(s) used to refine structure: *SHELXL97* (Sheldrick, 2008 ▶); molecular graphics: *SHELXTL* (Sheldrick, 2008 ▶); software used to prepare material for publication: *SHELXTL*.

## Supplementary Material

Crystal structure: contains datablock(s) I, global. DOI: 10.1107/S1600536812023008/hb6797sup1.cif

Structure factors: contains datablock(s) I. DOI: 10.1107/S1600536812023008/hb6797Isup2.hkl

Additional supplementary materials:  crystallographic information; 3D view; checkCIF report

## Figures and Tables

**Table 1:** Selected bond lengths (Å)

Fe1—Cl1′	2.178 (4)
Fe1—Cl2′	2.211 (7)
Fe1—Cl3′	2.256 (5)
Fe1—Cl4	2.1821 (17)

**Fig. 1.**
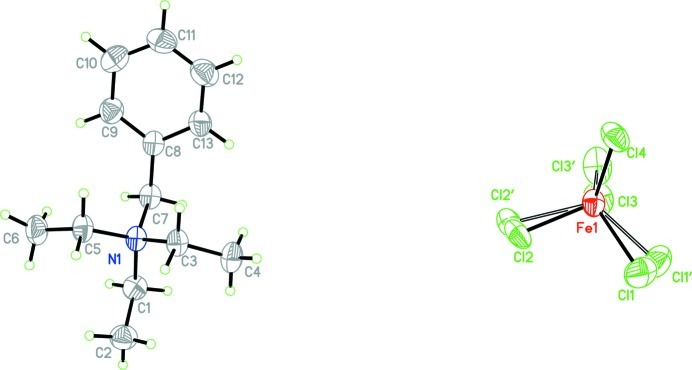
The molecular structure of the title compound, showing 30% probability displacement ellipsoids.

## supplementary crystallographic information

### Comment

In our lab, we has been exploring simple molecular-ionic componds which have potential phase-transition properties. (Zhang *et al.*, 2010). Herein, the title compound has been synthesized and its crystal structure is reported.

The ions of the title compound,(C_13_H_22_N^+^)^.^ FeCl^-^_4_ crystallize in the orthorhombic *Pbca* space group, an asymmetric unit consists of a tetrachloroferrate anion unit, which contains three disorded Cl1,Cl2,Cl3 atoms with a ratio in 1: 1, and benzyltriethylammonium cations (Sheme 1). (Fig 1). In the anion,the bond distances of Fe–Cl being in the range of 2.083 (7)–2.210 (8)? and the bond angles of Cl–Fe–Cl being in the range of 93.9 (7)–124.7 (4) °, while the bond distances of disorded Fe—Cl being in the range of 2.178 (4)–2.211 (7)? and the bond angles of disoeded Cl–Fe–Cl range from 90.7 to 127.9 °, which means a largely deviation to the ideal structure.In the structure,the benzyltriethylammonium cations interact with the FeCl^-^_4_anion through non-covalent interaction-static attracting forces like Coulomb and Van der Waals forces to complete a network structure.

### Experimental

In room temperature benzyltriethylammoniumchlorine (10 mmol, 2.28 g) were dissolved in 30 ml water, then a solution with FeCl_3_.6H_2_O (5 mmol, 1.35 g) and excessivehydrochloric acidwas dropped slowly into the previous solution with properly sirring. An orange solid appeared immediately and the solid was collected by filtration. Orange blocks were obtained by the slow evaporation of the above filtrate after a week in air.

The dielectric constant of the compound as a function of temperature indicates that the permittivity is basically temperature-independent (ε = C/(T–T_0_)), suggesting that this compound is not ferroelectric or there may be no distinct phase transition occurring within the measured temperature (below the melting point).

### Refinement

H atoms were placed in calculated positions(C—H = 0.93 Å for C*sp^2^* atoms and C—H = 0.96 Å and 0.97 Å for C*sp^3^* atoms), assigned fixed *U*_iso_ values [*U*_iso_ = 1.2*U*eq(C*sp^2^*/N) and 1.5*U*eq(C*sp^3^*)] and allowed to ride.

### Crystal data

**Table:**

(C_13_H_22_N)[FeCl_4_]	*F*(000) = 1608
*M**_r_* = 389.97	*D*_x_ = 1.376 Mg m^−^^3^
Orthorhombic, *P**b**c**a*	Mo *K*α radiation, λ = 0.71073 Å
Hall symbol: -P 2ac 2ab	θ = 3.0–26°
*a* = 15.514 (3) Å	µ = 1.36 mm^−^^1^
*b* = 15.021 (3) Å	*T* = 293 K
*c* = 16.155 (3) Å	Block, orange
*V* = 3764.7 (13) Å^3^	0.26 × 0.24 × 0.20 mm
*Z* = 8	

### Data collection

**Table:**

Rigaku Mercury2 CCD diffractometer	3691 independent reflections
Radiation source: fine-focus sealed tube	2364 reflections with *I* > 2σ(*I*)
Graphite monochromator	*R*_int_ = 0.082
Detector resolution: 13.6612 pixels mm^-1^	θ_max_ = 26.0°, θ_min_ = 3.0°
CCD_Profile_fitting scans	*h* = −19→19
Absorption correction: multi-scan (*CrystalClear*; Rigaku, 2005)	*k* = −18→18
*T*_min_ = 0.655, *T*_max_ = 0.734	*l* = −19→19
32945 measured reflections	

### Refinement

**Table:**

Refinement on *F*^2^	Secondary atom site location: difference Fourier map
Least-squares matrix: full	Hydrogen site location: inferred from neighbouring sites
*R*[*F*^2^ > 2σ(*F*^2^)] = 0.068	H-atom parameters constrained
*wR*(*F*^2^) = 0.170	*w* = 1/[σ^2^(*F*_o_^2^) + (0.0625*P*)^2^ + 5.6146*P*] where *P* = (*F*_o_^2^ + 2*F*_c_^2^)/3
*S* = 1.04	(Δ/σ)_max_ = 0.009
3691 reflections	Δρ_max_ = 0.73 e Å^−^^3^
204 parameters	Δρ_min_ = −0.38 e Å^−^^3^
156 restraints	Extinction correction: *SHELXL97* (Sheldrick, 2008), Fc^*^=kFc[1+0.001xFc^2^λ^3^/sin(2θ)]^-1/4^
Primary atom site location: structure-invariant direct methods	Extinction coefficient: 0.0092 (7)

### Special details

**Table:**

Geometry. All e.s.d.'s (except the e.s.d. in the dihedral angle between two l.s. planes) are estimated using the full covariance matrix. The cell e.s.d.'s are taken into account individually in the estimation of e.s.d.'s in distances, angles and torsion angles; correlations between e.s.d.'s in cell parameters are only used when they are defined by crystal symmetry. An approximate (isotropic) treatment of cell e.s.d.'s is used for estimating e.s.d.'s involving l.s. planes.
Refinement. Refinement of *F*^2^ against ALL reflections. The weighted *R*-factor *wR* and goodness of fit *S* are based on *F*^2^, conventional *R*-factors *R* are based on *F*, with *F* set to zero for negative *F*^2^. The threshold expression of *F*^2^ > σ(*F*^2^) is used only for calculating *R*-factors(gt) *etc*. and is not relevant to the choice of reflections for refinement. *R*-factors based on *F*^2^ are statistically about twice as large as those based on *F*, and *R*- factors based on ALL data will be even larger.

### Fractional atomic coordinates and isotropic or equivalent isotropic displacement parameters (Å^2^)

**Table:**

	*x*	*y*	*z*	*U*_iso_*/*U*_eq_	Occ. (<1)
C1	0.7598 (3)	0.8752 (4)	0.3510 (3)	0.0664 (14)	
H1A	0.8189	0.8604	0.3369	0.080*	
H1B	0.7231	0.8513	0.3078	0.080*	
C2	0.7509 (4)	0.9749 (4)	0.3512 (4)	0.0816 (17)	
H2A	0.6920	0.9906	0.3619	0.122*	
H2B	0.7679	0.9980	0.2982	0.122*	
H2C	0.7871	0.9996	0.3935	0.122*	
C3	0.6454 (3)	0.8524 (3)	0.4582 (3)	0.0585 (13)	
H3A	0.6316	0.8189	0.5078	0.070*	
H3B	0.6437	0.9150	0.4726	0.070*	
C4	0.5764 (3)	0.8349 (4)	0.3949 (4)	0.0789 (17)	
H4A	0.5873	0.8700	0.3464	0.118*	
H4B	0.5212	0.8506	0.4175	0.118*	
H4C	0.5765	0.7729	0.3804	0.118*	
C5	0.7951 (3)	0.8604 (4)	0.5021 (3)	0.0677 (14)	
H5A	0.7825	0.8254	0.5511	0.081*	
H5B	0.7813	0.9220	0.5146	0.081*	
C6	0.8915 (3)	0.8539 (4)	0.4842 (4)	0.0879 (19)	
H6A	0.9040	0.7971	0.4597	0.132*	
H6B	0.9232	0.8601	0.5349	0.132*	
H6C	0.9079	0.9004	0.4466	0.132*	
C7	0.7465 (3)	0.7295 (3)	0.4183 (3)	0.0590 (13)	
H7A	0.7085	0.7123	0.3733	0.071*	
H7B	0.8051	0.7176	0.4006	0.071*	
C8	0.7268 (3)	0.6716 (3)	0.4920 (3)	0.0574 (12)	
C9	0.7909 (4)	0.6451 (4)	0.5460 (4)	0.0751 (15)	
H9	0.8470	0.6648	0.5377	0.090*	
C10	0.7728 (5)	0.5891 (4)	0.6129 (4)	0.0880 (18)	
H10	0.8164	0.5715	0.6488	0.106*	
C11	0.6901 (5)	0.5607 (4)	0.6247 (4)	0.0864 (18)	
H11	0.6771	0.5245	0.6697	0.104*	
C12	0.6270 (4)	0.5847 (4)	0.5717 (5)	0.0929 (19)	
H12	0.5711	0.5642	0.5800	0.111*	
C13	0.6447 (3)	0.6390 (4)	0.5060 (4)	0.0738 (15)	
H13	0.6005	0.6544	0.4698	0.089*	
Cl1	−0.0663 (7)	0.7414 (8)	0.2546 (8)	0.116 (3)	0.344 (11)
Cl2	0.1300 (9)	0.7070 (9)	0.3047 (8)	0.089 (3)	0.344 (11)
Cl2'	0.1493 (4)	0.6778 (5)	0.2948 (5)	0.0878 (15)	0.656 (11)
Cl3	0.0269 (6)	0.5423 (8)	0.1865 (6)	0.109 (2)	0.344 (11)
Cl4	−0.02775 (10)	0.58556 (13)	0.41086 (9)	0.0936 (6)	
Cl1'	−0.0875 (3)	0.7000 (5)	0.2274 (3)	0.1154 (16)	0.656 (11)
Cl3'	0.0285 (3)	0.4954 (5)	0.2216 (4)	0.1169 (19)	0.656 (11)
Fe1	0.01578 (5)	0.62672 (6)	0.28825 (4)	0.0672 (3)	
N1	0.7371 (2)	0.8292 (3)	0.4325 (2)	0.0527 (10)	

### Atomic displacement parameters (Å^2^)

**Table:**

	*U*^11^	*U*^22^	*U*^33^	*U*^12^	*U*^13^	*U*^23^
C1	0.065 (3)	0.081 (4)	0.053 (3)	0.003 (3)	0.013 (2)	0.000 (3)
C2	0.085 (4)	0.081 (4)	0.079 (4)	0.000 (3)	0.004 (3)	0.010 (3)
C3	0.045 (2)	0.064 (3)	0.066 (3)	0.008 (2)	0.007 (2)	−0.019 (3)
C4	0.053 (3)	0.094 (4)	0.090 (4)	0.007 (3)	−0.007 (3)	−0.019 (3)
C5	0.059 (3)	0.082 (4)	0.062 (3)	−0.009 (3)	−0.006 (2)	−0.014 (3)
C6	0.052 (3)	0.109 (5)	0.103 (5)	−0.011 (3)	−0.006 (3)	0.004 (4)
C7	0.053 (3)	0.071 (3)	0.053 (3)	0.005 (2)	0.009 (2)	−0.014 (2)
C8	0.051 (3)	0.066 (3)	0.055 (3)	0.005 (2)	0.005 (2)	−0.013 (2)
C9	0.065 (3)	0.088 (4)	0.072 (4)	0.002 (3)	0.002 (3)	0.000 (3)
C10	0.099 (5)	0.096 (5)	0.070 (4)	0.011 (4)	−0.012 (3)	0.001 (3)
C11	0.105 (5)	0.082 (4)	0.072 (4)	−0.011 (4)	0.015 (4)	0.006 (3)
C12	0.080 (4)	0.091 (5)	0.108 (5)	−0.006 (4)	0.016 (4)	0.010 (4)
C13	0.056 (3)	0.081 (4)	0.085 (4)	−0.002 (3)	0.003 (3)	0.003 (3)
Cl1	0.099 (5)	0.130 (6)	0.120 (5)	0.050 (4)	0.052 (4)	0.027 (4)
Cl2	0.095 (6)	0.112 (6)	0.060 (4)	−0.056 (4)	−0.003 (4)	0.003 (4)
Cl2'	0.079 (2)	0.110 (4)	0.075 (2)	−0.026 (2)	0.0146 (16)	0.000 (2)
Cl3	0.125 (4)	0.123 (5)	0.080 (4)	−0.015 (4)	−0.004 (4)	−0.042 (4)
Cl4	0.0898 (11)	0.1249 (14)	0.0659 (9)	−0.0371 (10)	0.0069 (8)	0.0092 (9)
Cl1'	0.096 (2)	0.153 (4)	0.097 (3)	0.017 (2)	−0.0180 (19)	0.018 (2)
Cl3'	0.103 (2)	0.142 (4)	0.105 (3)	−0.004 (2)	0.010 (2)	−0.063 (3)
Fe1	0.0567 (5)	0.0958 (7)	0.0492 (4)	−0.0014 (4)	0.0059 (3)	−0.0069 (4)
N1	0.046 (2)	0.064 (3)	0.048 (2)	0.0034 (18)	0.0056 (17)	−0.0143 (19)

### Geometric parameters (Å, º)

**Table:**

C1—C2	1.503 (8)	C7—N1	1.522 (6)
C1—N1	1.528 (6)	C7—H7A	0.9700
C1—H1A	0.9700	C7—H7B	0.9700
C1—H1B	0.9700	C8—C9	1.381 (7)
C2—H2A	0.9600	C8—C13	1.384 (7)
C2—H2B	0.9600	C9—C10	1.397 (8)
C2—H2C	0.9600	C9—H9	0.9300
C3—C4	1.505 (7)	C10—C11	1.366 (9)
C3—N1	1.521 (5)	C10—H10	0.9300
C3—H3A	0.9700	C11—C12	1.350 (9)
C3—H3B	0.9700	C11—H11	0.9300
C4—H4A	0.9600	C12—C13	1.367 (8)
C4—H4B	0.9600	C12—H12	0.9300
C4—H4C	0.9600	C13—H13	0.9300
C5—N1	1.516 (6)	Cl1—Fe1	2.210 (8)
C5—C6	1.526 (7)	Cl2—Fe1	2.160 (12)
C5—H5A	0.9700	Cl3—Fe1	2.083 (7)
C5—H5B	0.9700	Fe1—Cl1'	2.178 (4)
C6—H6A	0.9600	Fe1—Cl2'	2.211 (7)
C6—H6B	0.9600	Fe1—Cl3'	2.256 (5)
C6—H6C	0.9600	Fe1—Cl4	2.1821 (17)
C7—C8	1.506 (7)		
			
C2—C1—N1	115.3 (4)	C13—C8—C7	121.3 (5)
C2—C1—H1A	108.4	C8—C9—C10	121.2 (5)
N1—C1—H1A	108.4	C8—C9—H9	119.4
C2—C1—H1B	108.4	C10—C9—H9	119.4
N1—C1—H1B	108.4	C11—C10—C9	118.9 (6)
H1A—C1—H1B	107.5	C11—C10—H10	120.5
C1—C2—H2A	109.5	C9—C10—H10	120.5
C1—C2—H2B	109.5	C12—C11—C10	120.7 (6)
H2A—C2—H2B	109.5	C12—C11—H11	119.7
C1—C2—H2C	109.5	C10—C11—H11	119.7
H2A—C2—H2C	109.5	C11—C12—C13	120.4 (6)
H2B—C2—H2C	109.5	C11—C12—H12	119.8
C4—C3—N1	116.1 (4)	C13—C12—H12	119.8
C4—C3—H3A	108.3	C12—C13—C8	121.5 (6)
N1—C3—H3A	108.3	C12—C13—H13	119.3
C4—C3—H3B	108.3	C8—C13—H13	119.3
N1—C3—H3B	108.3	Cl3—Fe1—Cl1'	90.7 (3)
H3A—C3—H3B	107.4	Cl3—Fe1—Cl2	111.6 (4)
C3—C4—H4A	109.5	Cl1'—Fe1—Cl2	112.2 (5)
C3—C4—H4B	109.5	Cl3—Fe1—Cl4	124.7 (4)
H4A—C4—H4B	109.5	Cl1'—Fe1—Cl4	108.99 (14)
C3—C4—H4C	109.5	Cl2—Fe1—Cl4	107.5 (4)
H4A—C4—H4C	109.5	Cl3—Fe1—Cl2'	99.9 (3)
H4B—C4—H4C	109.5	Cl1'—Fe1—Cl2'	122.4 (3)
N1—C5—C6	114.9 (4)	Cl2—Fe1—Cl2'	14.5 (4)
N1—C5—H5A	108.5	Cl4—Fe1—Cl2'	110.2 (2)
C6—C5—H5A	108.5	Cl3—Fe1—Cl1	109.1 (4)
N1—C5—H5B	108.5	Cl1'—Fe1—Cl1	21.8 (3)
C6—C5—H5B	108.5	Cl2—Fe1—Cl1	93.9 (7)
H5A—C5—H5B	107.5	Cl4—Fe1—Cl1	105.4 (2)
C5—C6—H6A	109.5	Cl2'—Fe1—Cl1	106.3 (5)
C5—C6—H6B	109.5	Cl3—Fe1—Cl3'	23.7 (2)
H6A—C6—H6B	109.5	Cl1'—Fe1—Cl3'	106.90 (18)
C5—C6—H6C	109.5	Cl2—Fe1—Cl3'	118.3 (4)
H6A—C6—H6C	109.5	Cl4—Fe1—Cl3'	102.3 (2)
H6B—C6—H6C	109.5	Cl2'—Fe1—Cl3'	104.12 (19)
C8—C7—N1	115.5 (4)	Cl1—Fe1—Cl3'	127.9 (4)
C8—C7—H7A	108.4	C5—N1—C3	106.3 (3)
N1—C7—H7A	108.4	C5—N1—C7	111.0 (4)
C8—C7—H7B	108.4	C3—N1—C7	110.9 (3)
N1—C7—H7B	108.4	C5—N1—C1	111.2 (4)
H7A—C7—H7B	107.5	C3—N1—C1	110.3 (4)
C9—C8—C13	117.2 (5)	C7—N1—C1	107.1 (3)
C9—C8—C7	121.4 (5)		
